# Gut microbiome-wide association study of depressive symptoms

**DOI:** 10.1038/s41467-022-34502-3

**Published:** 2022-12-06

**Authors:** Djawad Radjabzadeh, Jos A. Bosch, André G. Uitterlinden, Aeilko H. Zwinderman, M. Arfan Ikram, Joyce B. J. van Meurs, Annemarie I. Luik, Max Nieuwdorp, Anja Lok, Cornelia M. van Duijn, Robert Kraaij, Najaf Amin

**Affiliations:** 1 Department of Internal Medicine, Erasmus Medical Center Rotterdam, Rotterdam, the Netherlands; 2 Department of Psychology, University of Amsterdam, Amsterdam, The Netherlands; 3 Department of Medical Psychology, Amsterdam University Medical Centers, Amsterdam, The Netherlands; 4 Department of Epidemiology, Erasmus MC University Medical Center Rotterdam, Rotterdam, The Netherlands; 5 Department of Epidemiology and Data Science, Amsterdam University Medical Centers, Amsterdam, The Netherlands; 6 Department of Internal and Vascular Medicine, Amsterdam University Medical Centers, Location AMC, Amsterdam, The Netherlands; 7 Department of Psychiatry, Amsterdam University Medical Centers, Location AMC, Amsterdam, The Netherlands; 8 Nuffield Department of population Health, Oxford University, Oxford, UK

**Keywords:** Microbiology, Microbiome

## Abstract

Depression is one of the most poorly understood diseases due to its elusive pathogenesis. There is an urgency to identify molecular and biological mechanisms underlying depression and the gut microbiome is a novel area of interest. Here we investigate the relation of fecal microbiome diversity and composition with depressive symptoms in 1,054 participants from the Rotterdam Study cohort and validate these findings in the Amsterdam HELIUS cohort in 1,539 subjects. We identify association of thirteen microbial taxa, including genera *Eggerthella, Subdoligranulum, Coprococcus, Sellimonas, Lachnoclostridium, Hungatella, Ruminococcaceae (UCG002, UCG003 and UCG005), LachnospiraceaeUCG001, Eubacterium ventriosum* and *Ruminococcusgauvreauiigroup*, and family *Ruminococcaceae* with depressive symptoms. These bacteria are known to be involved in the synthesis of glutamate, butyrate, serotonin and gamma amino butyric acid (GABA), which are key neurotransmitters for depression. Our study suggests that the gut microbiome composition may play a key role in depression.

## Abstract (web summary)

Here, the authors analyze the relation of fecal microbiota diversity and composition with depressive symptoms in 1,054 participants from the Rotterdam Study cohort and in 1,539 subjects of the Amsterdam HELIUS cohort, finding associations with bacteria known to be involved in the synthesis of key neurotransmitters for depression.

## Introduction

Depression is one of the most common mental disorders experienced worldwide with an average lifetime prevalence of 11–15%^1^. The prevalence has doubled and, in some countries, even tripled during the COVID-19 pandemic^2^. Yet, depression is also one of the most common and poorly understood diseases courtesy of its elusive pathogenesis. Treatment options are sub-optimal with most antidepressants performing only marginally better than placebo^3,4^ with additional costs of having side effects ranging from minor cognitive complaints to even suicide^5^. The low to moderate heritability^6^ and the small effects of genetic variants (odds ratio <1.05) identified in large genome-wide association studies (GWAS) of depression^7^ entails the need to go beyond genetics in the search of molecular biomarkers of depression.

Evidence is accumulating that gut microbiota may influence brain activity and behavior via neural and humoral pathways^8,9^ and may have translational applications in the treatment of neuropsychiatric disorders^10–12^. Several animal studies suggest that gut microbiota might have impact on the neurobiological features of depression^13–21^. Fecal microbiota transplantation of either stressed or obese animals to control animals showed significant alteration of anxiety^22^. Kelly et al.^23^ showed that transferring gut microbiota of depressed human patients to germfree rats induces behavioral and physiological features characteristic of depression in the recipient animals suggesting that gut microbiota may be involved in causal pathways leading to depression. Another study showed that pre- and probiotic consumption positively affects mood and anxiety in humans^24^. There have been very few studies systematically exploring the association between gut microbiome and depression in humans^25^. Further, the existing studies are based on very small samples (<60 cases), lacking statistical power to detect robust and reproducible associations. The most recent study including 121 cases reported depletion of butyrate producing bacteria (*Coprococcus* and *Dialister*) in individuals with depression^26^. However, these studies did not adjust for confounders including life style factors and medication use^25^, which are known to modify the gut microbiome^27^. A parallel study investigated the association of the gut microbiome with depressive symptoms in the multiethnic HELIUS cohort comprising of six different ethnic groups. This study has identified genera/species belonging to the families *Christensencellaceae*, *Lachnospiraceae*, and *Ruminococcaceae* consistently associated with depressive symptoms across ethnicities, taking a wide range of confounders into account [NCOMMS-21-20669B]. Thus far, most consistent associations have been reported for genera *Eggerthella*, *Coproccocus*, *Subdoligranulum*, *Mitsuokella*, *Paraprevotella*, *Sutterella* and family Prevotellaceae^28^. However, the results of existing studies are conflicting with little overlap asking for larger and more carefully designed studies^28^.

Here, we study the effect of gut microbiome diversity and composition on depression scores in 1,133 individuals from the Rotterdam Study while controlling for lifestyle factors and medication use. The analyses were replicated in the native Dutch participants of the HELIUS cohort (N = 1,539). Finally, we performed Mendelian Randomization (MR) to elucidate causal relationships between the identified microbiota and major depression.

## Results

### Microbiome association analysis reveals association of thirteen taxa with depressive symptoms

The cohort characteristics are provided in Table 1. After exclusion of individuals using antidepressants and non-European subjects, 1,054 samples from RS and 1,539 samples from the HELIUS-study were included in the analyses (Table 1). The resulting microbiome data consisted of 17 phyla (for both cohorts), 33 classes for RS and 36 classes for HELIUS, 59 orders in RS and 61 orders for HELIUS, 116 families for RS and 108 families for HELIUS, and 439 genera for RS and 418 genera for HELIUS. In both cohorts, microbiome was dominated by phyla Firmicutes (77% in RS and 70% in HELIUS), Bacteroidetes (13% in RS and 21% in HELIUS), Actinobacteria (0.42% in RS and 0.42% in HELIUS) and Proteobacteria (0.48% in RS and 0.22% in HELIUS).

**Table 1:** Descriptive statistics of the study populations

	RS	HELIUS
Age: mean(±SD; range)	56 (±5.9; 45–87)	51 (±12.8; 19–71)
Sex (female%)	56%	49%
BMI: mean(±SD; range)	27 (±4.4; 16–51)	26 (±4.4; 16–53)
Smoking (current, ever, never)	(137, 533, 384)	(305, 664, 568)
Antidepressants (Yes)	79	66
Depression Score mean(±SD; range)	4.7 (±6.2; 0–49)	3 (±3.6; 0–24)

Descriptive statistics of the Rotterdam Study (N = 1054) and HELIUS (N = 1539) cohorts.

Alpha diversity was negatively associated with depressive symptoms in both RS (Shannon index; beta = −1.57, p value = 1.5 × 10^−03^) and HELIUS cohorts (Shannon index; beta = −0.64, *p* value = 2.84 × 10^−02^). Beta diversity showed significant association with depressive symptoms in RS (Permanova; *R*^2^ = 0.003, *p* value = 0.001) but not in the HELIUS cohort (*R*^2^ = 0.0005, *p* value = 0.51).

**Fig. 1:**
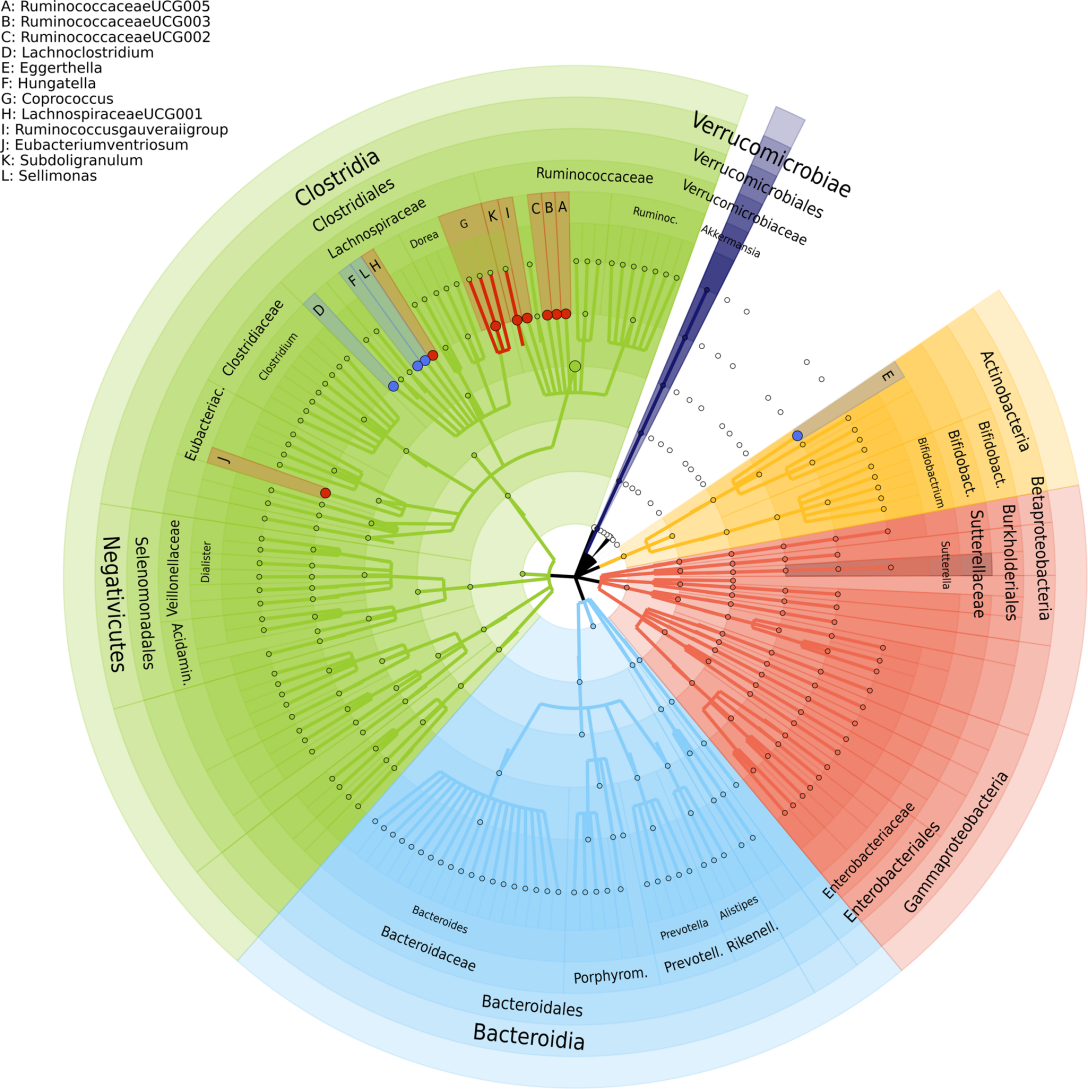
The taxonomic tree showing the 13 genera associated with depressive symptoms. Red dots depict negatively associated genera with depressive symptoms and blue ones depict positively associated genera with depressive symptoms. The outer most layer depicts the phylum level followed by class, order, family and genus levels.

At taxonomic level, 24 genera, three microbial families, one class, two orders and a phylum were significantly (false discovery rate (FDR) < 5%) associated with depressive symptoms in the Rotterdam Study (Table 2, Source Data). We replicated these results in the HELIUS cohort for 12 genera, which were associated with depressive symptoms scores in the same direction (Table 2, Fig. 1). These include *Sellimonas, Eggerthella, Ruminococcaceae (UCG002, UCG003, UCG005), Coprococcus3, Lachnoclostridium, Hungatella, LachnospiraceaeUCG001, Ruminococcusgauvreauiigroup, Eubacterium ventriosum* and *Subdoligranulum*. Of the three microbial families significantly associated with depressive symptoms in RS, only family Ruminococcaceae was significantly associated with depressive symptoms in the HELIUS cohort. The direction of association was consistent for all associated taxa across both cohorts and the meta-analysis of results from both cohorts improved association p-values (Table 2). Of the 12 significantly associated genera 10 belong to the families Ruminococcaceae and Lachnospiraceae. All significantly associated genera belonging to the family Ruminococcaceae were depleted in those with higher depressive symptoms (Fig. 1). While most of the significantly associated genera within family Lachnospiraceae were increased in those reporting higher depressive symptoms (Fig. 1).

**Table 2:** Microbiota significantly associated with depressive symptoms

Taxon	Rotterdam Study (N = 1054)				HELIUS (N = 1539)			Meta-analysis		
	Beta	Se	p	fdr	Beta	Se	P-value	N	Zscore	*P*-value
family.Christensenellaceae.id.1866	−0.59	0.12	4.56E–07	8.04E–05	−0.10	0.06	1.32E–01	2593	−1.88	5.96E–02
*genus.ChristensenellaceaeR7group.id.11283*	−0.55	0.11	3.31E–07	8.04E–05	−0.10	0.06	1.30E–01	2593	−4.42	9.80E–06
***genus.RuminococcaceaeUCG005.id.11363***	**−0.59**	**0.13**	**1.36E–05**	**1.57E–03**	**−0.12**	**0.07**	**7.62E–02**	**2593**	**−4.14**	**3.48E–05**
*genus.RuminococcaceaeUCG010.id.11367*	−0.50	0.12	1.77E–05	1.57E–03	−0.07	0.06	2.08E–01	2593	−3.71	2.11E–04
***family.Ruminococcaceae.id.2050***	**−1.59**	**0.38**	**2.51E–05**	**1.77E–03**	**−0.52**	**0.24**	**3.11E–02**	**2593**	**−4.35**	**1.38E–05**
*genus.Coprococcus2.id.11302*	−0.29	0.08	2.74E–04	1.03E–02	−0.02	0.04	5.79E–01	2593	−2.75	6.02E–03
*genus.FamilyXIIIAD3011group.id.11293*	−0.74	0.20	2.56E–04	1.03E–02	−0.06	0.10	5.78E–01	2593	−2.76	5.78E–03
***genus.Lachnoclostridium.id.11308***	**0.57**	**0.16**	**3.11E–04**	**1.03E–02**	**0.27**	**0.11**	**1.80E–02**	**2593**	**4.12**	**3.76E–05**
***genus.RuminococcaceaeUCG002.id.11360***	**−0.42**	**0.12**	**3.21E–04**	**1.03E–02**	**−0.16**	**0.07**	**2.33E–02**	**2593**	**−4.04**	**5.30E–05**
***genus.RuminococcaceaeUCG003.id.11361***	**−0.51**	**0.15**	**4.60E–04**	**1.33E–02**	**−0.25**	**0.08**	**3.10E–03**	**2593**	**−4.51**	**6.42E–06**
*class.Mollicutes.id.3920*	−0.39	0.12	8.63E–04	1.38E–02	−0.01	0.05	8.04E–01	2593	−2.32	2.06E–02
**genus.Eggerthella.id.819**	**0.65**	**0.19**	**6.28E–04**	**1.38E–02**	**0.31**	**0.12**	**1.18E–02**	**2593**	**4.12**	**3.80E–05**
**genus.Hungatella.id.11306**	**0.77**	**0.22**	**6.73E–04**	**1.38E–02**	**0.34**	**0.14**	**1.34E–02**	**2593**	**4.07**	**4.65E–05**
*genus.Ruminiclostridium6.id.11356*	−0.36	0.11	6.23E–04	1.38E–02	−0.01	0.05	9.06E–01	2593	−2.27	2.31E–02
*genus.RuminococcaceaeUCG014.id.11371*	−0.29	0.08	5.70E–04	1.38E–02	−0.05	0.05	3.16E–01	2593	−2.97	2.99E–03
*order.MollicutesRF9.id.11579*	−0.40	0.12	8.28E–04	1.38E–02	0.00	0.05	9.55E–01	2593	−2.18	2.96E–02
*phylum.Tenericutes.id.3919*	−0.39	0.12	8.63E–04	1.38E–02	−0.01	0.05	8.04E–01	2593	−2.32	2.06E–02
***genus.Coprococcus3.id.11303***	**−0.43**	**0.13**	**9.09E–04**	**1.39E–02**	**−0.29**	**0.09**	**1.20E–03**	**2593**	**−4.61**	**4.04E–06**
***genus.LachnospiraceaeUCG001.id.11321***	**−0.52**	**0.16**	**1.01E–03**	**1.48E–02**	**−0.16**	**0.07**	**2.34E–02**	**2593**	**−3.84**	**1.21E–04**
*family.Micrococcaceae.id.636*	1.53	0.47	1.26E–03	1.71E–02	−0.14	0.16	3.75E–01	2593	2.92	3.55E–03
***genus..Ruminococcusgauvreauiigroup.id.11342***	**−0.34**	**0.11**	**1.54E–03**	**2.01E–02**	**−0.13**	**0.07**	**4.90E–02**	**2593**	**−3.54**	**4.06E–04**
***genus..Eubacteriumventriosumgroup.id.11341***	**−0.49**	**0.16**	**2.09E–03**	**2.47E–02**	**−0.17**	**0.08**	**4.20E–02**	**2593**	**−3.53**	**4.19E–04**
***genus.Sellimonas.id.14369***	**0.57**	**0.18**	**2.10E–03**	**2.47E–02**	**0.54**	**0.13**	**3.55E–05**	**2593**	**5.15**	**2.65E–07**
*genus.Rothia.id.646*	1.47	0.48	2.21E–03	2.52E–02	0.32	0.23	1.66E–01	2593	3.02	2.55E–03
*order.Micrococcales.id.510*	1.41	0.47	2.58E–03	2.84E–02	−0.21	0.15	1.68E–01	2593	2.96	3.09E–03
*genus.LachnospiraceaeNK4A136group.id.11319*	−0.41	0.14	2.80E–03	2.99E–02	−0.08	0.08	3.48E–01	2593	−2.63	8.56E–03
**genus.Subdoligranulum.id.2070**	**−0.44**	**0.15**	**3.36E–03**	**3.48E–02**	**−0.28**	**0.09**	**1.66E–03**	**2593**	**−4.29**	**1.77E–05**
*genus.RuminococcaceaeNK4A214group.id.11358*	−0.34	0.12	4.66E–03	4.70E–02	−0.04	0.06	5.73E–01	2593	−2.24	2.52E–02
*genus.PrevotellaceaeUCG001.id.11186*	−0.49	0.17	4.80E–03	4.71E–02	−0.02	0.08	8.38E–01	2593	−1.96	5.06E–02

Significantly associated taxa with depressive symptom levels in both Rotterdam Study (N = 1,054) and HELIUS (N = 1,539) cohorts. Linear regression models were used adjusting for several life-style confounders (such as smoking and alcohol use) and medical confounders (such as PPI, metformin). The taxa at genus level are in italic face and taxa that remain significant in both discovery and replication cohort and after the meta-analysis are highlighted in bold face. The taxa Data for the regression analysis of the Rotterdam Study are provided as a Source Data file.

Random forest analysis with RS as the training cohort and HELIUS as the testing cohort revealed *RuminococcaceaeUCG005* as the most important genus in predicting depressive symptoms (Source Data), showing the highest percentage increase in mean squared error (%incMSE) in out of bag analysis. Other important predictors of depressive symptoms include *ChristensenellaceaeR7group, Lachnoclostridium, Eggerthella, Sellimonas*, and *Hungatella*, which overlap with the findings of the linear regression analysis in this study (Source Data). Further, important predictors identified by random forest analysis include *Roseburia*, *Streptococcus*, *Bacteroides*, *Anaerotruncus*, *Dorea*, *Blautia*, *Veillonella*, *Desulfovibrio*, *Anaerostipes* and *Bifidobacterium*, which replicate associations reported earlier^28^.

### Mendelian Randomization (MR) analysis identifies a causal link between major depression and Eggerthella

Results of MR analysis are provided in the Source Data. With major depression as the exposure, *Eggerthella*, showed significant MR results under the IVW method (effect = 0.237, *p value* = 0.027) (Source Data). Tests for heterogeneity and horizontal pleiotropy were negative for *Eggerthella* (Supplementary Data 1, Supplementary Data 2). Further the effect estimates for *Eggerthella* was also consistent with the findings of this study, i.e., increase in the abundance of *Eggerthella* in those with higher depressive symptoms. Interestingly, the Steiger test for directionality suggests that *Eggerthella* is more likely to be causally associated with MDD (Supplementary Data 3). With microbiome as exposure, significant MR was observed for genus *Sellimonas* under the IVW method (effect = −0.046, *p-value* = 5.5*10^−04^) but effect estimate was inconsistent with the findings of our study (Supplementary Data 3).

Among the 87 depression-associated SNPs^7^ significant association was observed for one SNP rs17641524 with the genus *Acidaminococcus* after correction for multiple testing (Supplementary Data 4). No significant association was observed for the MDD GRS (Supplementary Data 5).

## Discussion

In this large study of 2593 individuals profiled for depressive symptoms and fecal microbiome, we identified 12 genera and 1 microbial family associated with depressive symptoms. These include genera *Sellimonas, Eggerthella, Ruminococcaceae (UCG002, UCG003, UCG005), Lachnoclostridium, Hungatella, Coprococcus, LachnospiraceaeUCG001, Ruminococcusgauvreauiigroup, Eubacterium ventriosum, Subdoligranulum* and family Ruminococcaceae*. Sellimonas, Eggerthella, Lachnoclostridium and Hungatella* were more abundant in individuals with higher depressive symptoms. All other taxa were depleted in depression. Alpha diversity was significantly associated with depressive symptoms in both discovery and replication cohorts.

The intestinal bacterial strains *Eggerthella, Subdoligranulum, Coprococcus* and *Ruminococcaceae* have been reported to be associated with major depression in earlier studies. *Eggerthella* has been consistently found to be increased in depression and anxiety cases in 8 studies^25,26,28^, which is in line with the findings of our study. MR analysis suggests a causal link between MDD and *Eggerthella*, which requires further investigation. Also in line with our findings *Subdoligranulum* and *Coprococcus* were consistently found to be depleted in individuals with generalized anxiety disorder and depression in several studies^28^. In a recent study Coproccocus was depleted in rats that exhibited depressed behavior upon fecal transplantation from depressed human subjects^29^, suggesting that Coproccocus may have a causal impact on depression. Both *Subdoligranulum* and *Coprococcus* are involved in the production of butyrate^26^ and *Subdoligranulum* was found to be increased in omega 3 rich diet^30^. A previous meta-analysis shows that omega 3 fatty acids, more specifically eicosapentaenoic acid (EPA) supplementation are beneficial for depression^31^. *Ruminococcaceae* at genus and family levels have been found to be depleted in cases of both uni- and bipolar depression^25,26,28,32–34^. A similar pattern is observed in the study by Bosch et al. [NCOMMS-21-20669B] with several genera belonging to the family Ruminococcaceae depleted in those reporting higher depressive symptoms, which is again consistent with the results of our study.

Other findings of this study that have previously not been reported include association with genera *Sellimonas*, *Lachnoclostridium*, *Hungatella*, *Eubacterium* v*entriosum*, *LachnospiraceaeUCG001*, and *Ruminococcusgauvreauiigroup*. *Sellimonas* and *Hungatella* were positively associated with depressive symptoms. *Sellimonas* is the most significant finding of this study. It belongs to the family Lachnospiraceae and phylum Firmicutes. Species belonging to *Sellimonas* have been reported to be increased in inflammatory diseases including ankylosing spondylitis, atherosclerosis and liver cirrhosis^35^. Further, increased abundance of *Sellimonas* have been observed after dysbiosis^36^. *Lachnoclostridium* belongs to the family Lachnospiraceae. Higher levels of *Lachnoclostridium* were associated with increased depressive symptoms in our study and also consistent with the findings of the Bosch et al. study [NCOMMS-21-20669B]. *Lachnoclostridium* has previously found to be depleted in other psychiatric disorders including schizophrenia^37^ and autism^38^ and in patients with gastrointestinal tract neoplasms^39^. *Hungatella* belongs to the family Clostridiaceae and phylum Firmicutes. It has previously been associated with paleolithic diet and is known to produce the precursor molecule for trimethylamine-N-oxide (TMAO)^40^. TMAO has been implicated in cardio-vascular and neurological diseases including depression^41,42^. *Eubacterium ventriosum* belongs to the family Eubacteriaceae and has been found to be significantly depleted after traumatic brain injury in mice^43^. Major depression is a frequent complication of traumatic brain injury^44^. In our study we also observed depletion of *Eubacterium ventriosum* with the increase in depressive symptoms, which fits well with association with traumatic brain injury. In human studies *Eubacterium ventriosum* was found to be slightly more abundant in obese individuals^45,46^. Obesity is one of the most prevalent somatic comorbidities of major depressive disorder^47,48^ and is partly attributed to a side effect of selective serotonin reuptake inhibitors (SSRI). However, in our study we excluded those using antidepressants and adjusted for BMI in the linear regression analysis thus our finding is independent of the association with body weight. *LachnospiraceaeUCG001*, at species level, was found to be associated with anhedonia in mice^49^. *Ruminococcusgauvreauii* belongs to the family Ruminococcaceae and at species level was found to be increased in atherosclerotic conditions^35^. Interestingly depression is known to be causally associated with atherosclerosis^50^. It may be worth to investigate the genera *Sellimonas* and *Ruminococcusgauvreauii* as potential mediators in the relationship between depression and atherosclerotic conditions.

Most identified microbiota in our study show potential involvement in the synthesis of glutamate and butyrate (see Supplementary Data of Valles-Colomer et al. 2019)^26^. *Eggerthella* is further involved in the synthesis of serotonin and gamma aminobutyric acid (GABA). Glutamate is widely distributed in the brain and a major excitatory synaptic neurotransmitter^51^. It is known to be involved in regulating neuroplasticity, learning and memory^52^. Glutamate levels in plasma, serum, cerebrospinal fluid and brain tissue have been associated with mood and psychotic disorders and suicide^53–58^. With increasing evidence of its role in the etiology of depressive disorders, glutamate is rapidly becoming the novel therapeutic target for depressive disorders. Ketamine, for instance, has been shown to increase glutamate signaling in rodents and humans^59,60^ and has shown to reduce depressive symptoms rapidly^61^. Glutamate plays a role as a neurotransmitter in the enteric nervous system, which sustains the reciprocal influence between the gastrointestinal tract and the central nervous system^8,62^. Butyrate on the hand is a short chain fatty acid and modulates biological responses of host gastrointestinal health by acting as a histone deacetylase inhibitor and binding to specific G protein-coupled receptors (GPCRs)^63^. Butyrate can affect the gut-brain axis by enhancing the cholinergic neurons via epigenetic mechanisms^64^ and can cross the blood brain barrier and activate the vagus nerve and hypothalamus^65,66^. Sodium butyrate has shown anti-depressant effects in animal models of depression and mania^67,68^. Serotonin and GABA are both important neurotransmitters relevant to depression. Evidence suggests that serotonin may be the key neurotransmitter to the gut-brain axis^17^. Enteric nervous system accounts for >90% of the body’s serotonin production where it is produced by enterochromaffin cells and in the neurons of the enteric nervous system^69^. The neuronal production of serotonin is most critical for the development and motility of the enteric nervous system, affecting neurogenesis and guiding development of neurons expressing dopamine and GABA^69–71^. Although serotonin produced by the gut cannot cross the blood-brain barrier^72^, it can affect the blood-brain barrier permeability, which can lead to inflammation of the brain^73^. Further, vagus nerve stimulation by the gut microbiota can alter concentration of serotonin, GABA and glutamate within the brain in animals and humans^42,74^ and germ-free male mice exhibit anxiety-like behaviors and altered serotonin abundance in the brain^14^. GABA is the main inhibitory neurotransmitter of the central nervous system that counterbalances the action of glutamate^75^. Low levels of GABA are linked to depression and mood disorders^75^. Animal studies show that gut microbiota can alter GABA activity in the brain through the vagus nerve^76^. While each of the metabolites mentioned above are highly relevant for depression, most are known to be unable to cross the blood-brain barrier. However, an increasing number of animal studies show that the peripheral production of neurotransmitters by the gut microbiome can alter brain chemistry and therefore influence mood and behavior^42^.

In the current study, we aimed to identify gut microbiota associated with depressive symptoms in the general population. The strengths of our study include a large sample, controlling for most known confounders including comorbid conditions, performing analysis in individuals free of anti-depressive medication and finally the use of quantitative depression scales. A large study consisting of 252,503 individuals from 68 countries showed that subthreshold depressive disorders produce significant decrements in health and do not qualitatively differ from full-blown episodes of depression^77^. Use of rating scales is thus more powerful in omics association studies^78^. There may have been a loss of statistical power as the depression assessment scales were different in the discovery and replication cohorts. Further, despite the use of the largest GWAS for both microbiome and depression, the MR analysis lacked power. There are 87 SNPs identified for depression, however, their effect on depression is small (individual odds ratio <1.05, combined odds ratio <2.0), which makes unlikely that the individual genetic variants show association with microbiome. For microbiome, there were no SNPs significantly associated at the genome-wide level. Therefore, we had to lower the threshold to 10^−05^ to identify at least more than one independent instrument for the identified microbiota. This limits the value of the MR. Another limitation of this study is using different methods for stool sampling and sequencing variable regions. These factors might influence the microbial profiles substantially. For example, reads generated by the V4 primer pair showed a higher alpha diversity of the gut microbial community than V1-V2 and V3-V4^79^. In addition, a recent study showed significant differences in bacterial composition that result from collecting stool samples using different stool collection methods compared to immediate freezing^80^. This may have a negative impact on statistical power. However, despite the differences there is a significant overlap and consistency in effect estimates between the discovery and the replication cohorts. The overlapping results of this study are, therefore, of greater importance, as they are consistent despite methodological differences. It is interesting to note that despite the fact that we replicate most of our findings in the European participants of the HELIUS cohort, there’s only partial overlap with the findings of the study by Bosch et al. [NCOMMS-21-20669B]. However, the lack of significant ethnic differences in that study suggests that non-replication between cohorts (as inferred by p-value testing) likely is in the realm of normal sample variation and coupled to small effect sizes, i.e., may (at least partially) reflect Type 2 statistical error. Another difference is the classification method used for taxonomic identification of bacteria. Bosch et al. uses Amplicon Sequence Variants in 3,211 participants from 6 ethnic groups [NCOMMS-21-20669B], while this study uses closed reference OTU clustering with the same SILVA database in only European participants. Nevertheless, irrespective of the above methodological differences reproduced associations are observed for *Lachnoclostridium*, *Coproccocus* and Ruminoccocaceae [NCOMMS-21-20669B] suggesting robust association of depressive symptoms with these taxa.

To summarize, we have identified several bacteria at genera level that might influence depression in humans. We confirm the association of *Eggerthella, Coprococcus*, *Subdoligranulum* and family Ruminococcaceae and identify bacteria including *Sellimonas, Lachnoclostridium, Hungatella, Ruminococcus, Subdoligranulum, LachnospiraceaeUCG001, Eubacterium ventriosum* and *Ruminococcusgauvreauiigroup*. These bacteria are involved in the synthesis of glutamate, butyrate, serotonin and GABA, which are the key neurotransmitters relevant for depression.

## Methods

### Study population

The discovery cohort includes 1054 participants from the Rotterdam Study who were not using anti-depressants at the time of assessment. The Rotterdam Study is a population-based cohort study from the well-defined Ommoord district within Rotterdam, The Netherlands. It is designed to investigate occurrence and determinants of diseases in the elderly^81^. Initially, the RS included 7,983 participants in 1990 who underwent an at-home interview, extensive physical examination at baseline and during follow-up examinations that occur every 3–4 years (RS-I). The RS was extended with two more cohorts in 2000 (RS-II) and 2005 (RS-III) and contains a total of 14,926 participants. In this study we used the data of individuals from the second follow up of the third Rotterdam Study cohort (RS-III-2) as these individuals were profiled for the gut microbiome. The Rotterdam Study (RS-III-2) consists of individuals of European background. The RS is approved by the Medical Ethics Committee of the Erasmus MC (registration number MEC 02.1015) and by the Dutch Ministry of Health, Welfare and Sport (Population Screening Act WBO, license number 1071272-159521-PG). The RS was entered into the Netherlands National Trial Register (NTR; www.trialregister.nl) and into the WHO International Clinical Trials Registry Platform (ICTRP; www.who.int/ictrp/network/primary/en/) under shared catalog number NTR6831. All participants provided written informed consent to participate in the study and to have their information obtained from treating physicians. Participants were not compensated for their participation.

The replication cohort included 1539 participants from the Healthy Life in an Urban Setting (HELIUS) cohort. The HELIUS cohort is a multiethnic cohort consisting of individuals of Dutch, Surinamese, Ghanaian, Turkish and Moroccan origin from Amsterdam. People in the age range of 18–70 years were randomly sampled, stratified by ethnic origin through the municipality register of Amsterdam. This register contains data on country of birth of citizens and of their parents, thus allowing for sampling based on the widely accepted Dutch standard indicator for ethnic origin^82^. The Dutch sample includes people who were born in the Netherlands and whose parents were born in the Netherlands. The current study used data from Dutch samples only. The Medical Ethics Committee of the Amsterdam UMC, location AMC approved the study protocols. Written informed consent was obtained from all participants, who were not compensated for their participation.

### Fecal sample collection and microbiome profiling

Detailed description on how the gut microbiome composition was generated at RS-III-2 (2012-2013) and in the HELIUS cohort are described elsewhere^83,84^. Briefly, in RS, participants were instructed to collect a stool sample at their home in sterile tubes and to send the sample by regular mail to the research location of Erasmus Medical Center (EMC), Rotterdam, the Netherlands. Upon arrival at Erasmus MC, samples were checked and stored at −20 °C. Samples, which were underway for more than 3 days, were excluded^84^. Subsequently, an automated stool DNA isolation kit (Diasorin, Saluggia, Italy) was used to isolate bacterial DNA from approximately 300 mg stool aliquot using a bead-beating step. The V3 and V4 hypervariable regions of the bacterial 16 S rRNA gene were amplified and sequenced on an Illumina MiSeq platform with the V3 kit (2 × 300 bp paired-end reads; Illumina).

Participants from the HELIUS-study were given a stool collection tube and requested to collect a stool sample and bring their samples to the research location within 6 h after collection and if not possible kept in their freezer overnight and bring it to the research location the next morning. At the research location, the samples were temporarily stored at −20 °C until daily transportation to the Amsterdam Medical Center (AMC), Amsterdam, the Netherlands, where the samples were checked and stored at −80 °C. Total genomic DNA was extracted from a 150 mg aliquot using a repeated bead beating method^85^. Briefly, fecal samples were bead beated twice and after each bead-beating cycle, samples were heated at 95 °C for 5 min. Supernatants from two extractions were pooled and DNA was purified using the QIAamp DNA Mini kit (QIAGEN Benelux B.V., Venlo, The Netherlands) on the QIAcube (QIAGEN) instrument using the procedure for human DNA analysis.

The composition of fecal microbiota was determined by sequencing the V4 region of the 16 S rRNA gene on a MiSeq system (Illumina) with 515 F and 806 R primers designed for dual indexing (42) and the V2 kit (2 × 250 bp paired-end reads; Illumina). Raw sequencing data from both cohorts were run through the same microbiome-profiling pipeline to harmonize the microbiome data.

Reads were subsampled at 10,000 reads per sample. Taxonomy was assigned using the standard profiling pipeline developed by the MiBioGen consortium^86^. Briefly, we implemented the 16 S data processing pipeline, which comprised closed reference OTU clustering without a de-noising step based on the naive Bayesian classifier from the Ribosomal Database Project (version 2.12) and the most recent (version 128), full, SILVA database. We only analyzed taxonomical results at genus and higher taxonomic levels. Alpha diversity indices such as species richness, Shannon index and Inverse Simpson were calculated at the genus-level. We calculated Bray-Curtis distances based on absolute abundance of microbial communities at genus level to measure beta-diversity. For single taxon analyses, taxa that were present in less than 3% of the sample size (each cohort separately) and taxa with read counts less than 0.005% of the total number of reads were excluded. Taxa abundances (absolute counts) were then log transformed (to the absolute values 1 was added before log-transformation).

### Depression assessment

In RS depressive symptoms were assessed using the 20-item version of the Center for Epidemiological Studies-Depression (CES-D) scale^87^. CES-D is a self-report measure of symptoms experienced during the prior week. It has been shown to be relatively stable over time and covers the major dimensions of depression including depressed mood, feelings of guilt and worthlessness, feelings of helplessness and hopelessness, psychomotor retardation, loss of appetite and sleep disturbance^88^. The total score ranges from 0 to 60, with higher scores indicating a greater burden of depressive symptoms. The CES-D detects current MDD cases with high sensitivity and specificity. We used the depression assessment from RS-III-2 (the same time as the collection of the feces).

For participants of the HELIUS cohort, depression was assessed using the Patient Health Questionnaire (PHQ-9) design^89^. PHQ-9 scores each of the DSM-IV criteria as “0” (not at all) to “3” (nearly every day). The total score ranges from 0 to 21, with higher scores indicating severity of depression. A PHQ-9 score of ≥10 has a sensitivity and specificity of 88% to detect major depression. Individuals with ethnic background other than Europeans and individuals using antidepressants were excluded.

### Statistics and reproducibility

Overall, no statistical method was used to predetermine sample size. No data were excluded from the analysis and the experiments were not randomized. The investigations were not blinded to allocation during experiments and outcome assessment.

#### Microbiome association analysis

To test the association of depressive symptom scores with alpha diversity and individual taxa we used linear regression models using depression scores as the outcome and alpha diversity and taxa (log+1 transformed) as independent variables adjusting for several covariates including sex, age, alcohol use, body mass index (BMI), smoking, medication use (proton pump inhibitors (PPI), metformin, lipid-lowering and antibiotics) and technical covariates including time in mail and batch (in case of RS cohort). Association of the depression scores with microbiome beta-diversity was performed using permutation analysis of variance (PERMANOVA) in R-package “vegan” using the same model as described above.

Results from the discovery and replication cohorts were combined in a meta-analysis using METAL software^90^. Since the depressive symptoms assessment scales were different in the discovery and replication cohorts, we used sample-size weighted meta-analysis to combine the results. Adjustment for multiple testing was performed using false discovery rate (FDR) using Benjamini-Hochberg correction.

Further, we performed a random forest regression analysis using Breiman’s random forest algorithm^91^ for regression implemented in the “randomForest” library of the R software. Random forest is a tree-based machine learning algorithm that captures non-linear relationships and can deal with highly correlated input data by leveraging the power of multiple decision trees in order to control overfitting problem. In particular, each tree in the ensemble is built from a bootstrapped sample from the training sample and each node of the tree works on a random subset of the total feature. For this analysis RS stool microbiome profiles were used as predictors and depression scores as response for the training data set, while the HELIUS stool microbiome profiles and depression scores as predictors and response as the test data. Hyperparameters of the model including number of trees (ntree = 500) and number of variables randomly sampled as candidates at each split (mtry = 100) were tuned to give the best performance based on the increase in mean square error (%IncMSE) calculated from out-of-bag samples. In addition, we set the number of times the out of bag data is permuted per tree for assessing variable importance to 100 (nPerm = 100).

#### Mendelian randomization (MR) analysis

To ascertain causal links between the identified microbiota and major depressive disorder (MDD) we performed two-sample MR analysis using the results of the largest genome-wide association studies of both microbiome and major depression^7,92^. For major depression we used genome-wide significant single nucleotide polymorphisms (SNPs) as instruments^7^ (Source Data). For microbiome there were none to a very few SNPs that were genome-wide significantly associated with the identified microbiota, so we used SNPs with a p-value <10^−05^ as instruments (Source Data). MR analysis was performed using the “TwoSampleMR” library^93^ of the R software. Linkage disequilibrium pruning of the SNPs was performed using the ‘clump_data’ option with the clump r2 of 0.01 to identify independent instruments. MR report was generated using the ‘mr_report’ option. This method reports results from the weighted median, simple and weighted mode, Inverse variance weighted (IVW) and Egger methods. Variance explained (R^2^) per instrument for both the exposures and the outcomes were generated using the ‘add_rsq’ option.

We further examined the microbiome-wide association of each of the 87 SNPs associated with depression using the microbiome GWAS summary statistics from Kurilshikov et al.^92^ to identify the gut microbiota associated with these SNPs. Finally, we tested the association of the genetic risk score combining the summary level data of the 87 SNPs for each microbiota in an unweighted genetic risk score using inverse-weighted method in the ‘rmeta’ package of R software.

### Reporting summary

Further information on research design is available in the Nature Portfolio Reporting Summary linked to this article.

## Supplementary information

Description of Additional Supplementary Files

Supplementary Data 1

Supplementary Data 2

Supplementary Data 3

Supplementary Data 4

Supplementary Data 5

Reporting Summary

STORMS Checklist

## Data Availability

All relevant data supporting the key findings of this study are available within the article and its supplementary files. Full results of the genus-level linear models (Fig. 1, and Table 2), Random forest analyses and Mendelian randomization are supplied as Source Data. Individual-level data of Rotterdam Study and HELIUS Study are not publicly available due to privacy regulations (GDPR). Also, raw 16 S sequencing data from the Rotterdam Study is not publicly available as sharing of participant data, either pseudo-anonymized or anonymized, was not part of the informed consent. Raw 16 S sequencing data from HELIUS participants is available through European Genome-Phenome archive (EGAD00001004106). Rotterdam Study data are available upon request to the data manager Frank van Rooij (f.vanrooij@erasmusmc.nl) and subject to local rules and regulations. This includes submitting a proposal to the management team of RS, where upon approval, analysis needs to be done on a local server with protected access, complying with GDPR regulations. Source data are provided with this paper.

## Source data

Source Data

## Code availability

The codes used for the analyses in this study are available at https://github.com/Djawad-Radj/Microbiome_Depression.
