# Supplementary material for: Gut microbiome-wide association study of depressive symptoms
Source: Nat Commun. 2022 Dec 6;13:7128. doi: 10.1038/s41467-022-34502-3 (PMC9726982; doi:10.1038/s41467-022-34502-3)
Supplement: Supplementary file 1 — Description of Additional Supplementary Files [file 41467_2022_34502_MOESM1_ESM.docx]

**Description of Additional Supplementary Files:**

**File name:** Supplementary Data 1

**Description:** Results of heterogeniety test in Mendelian Randomization analysis.

**File name:** Supplementary Data 2

**Description:** Results for the test of Pleiotropy in MR.

**File name:** Supplementary Data 3

**Description:** Results of test of directionality in MR.

**File name:** Supplementary Data 4

**Description:** Association results of MDD SNPs to microbial taxa.

**File name:** Supplementary Data 5

**Description:** Association results of MDD GRS with microbial taxa.
