# Supplementary material for: Gut microbiome-wide association study of depressive symptoms
Source: Nat Commun. 2022 Dec 6;13:7128. doi: 10.1038/s41467-022-34502-3 (PMC9726982; doi:10.1038/s41467-022-34502-3)
Supplement: Supplementary file 7 — Reporting Summary [file 41467_2022_34502_MOESM7_ESM.pdf]

nature research

Corresponding author(s): Najaf Amin, Robert Kraaij

Last updated by author(s): Jun 7, 2021

Reporting Summary

Nature Research wishes to improve the reproducibility of the work that we publish. This form provides structure for consistency and transparency in reporting. For further information on Nature Research policies, see our Editorial Policies and the Editorial Policy Checklist.

Statistics

For all statistical analyses, confirm that the following items are present in the figure legend, table legend, main text, or Methods section.

n/a	Confirmed	
☐	☒	The exact sample size (n) for each experimental group/condition, given as a discrete number and unit of measurement
☐	☒	A statement on whether measurements were taken from distinct samples or whether the same sample was measured repeatedly
☐	☒	The statistical test(s) used AND whether they are one- or two-sided<br>*Only common tests should be described solely by name; describe more complex techniques in the Methods section.*
☐	☒	A description of all covariates tested
☐	☒	A description of any assumptions or corrections, such as tests of normality and adjustment for multiple comparisons
☐	☒	A full description of the statistical parameters including central tendency (e.g. means) or other basic estimates (e.g. regression coefficient) AND variation (e.g. standard deviation) or associated estimates of uncertainty (e.g. confidence intervals)
☐	☒	For null hypothesis testing, the test statistic (e.g. F, t, r) with confidence intervals, effect sizes, degrees of freedom and P value noted<br>*Give P values as exact values whenever suitable.*
☒	☐	For Bayesian analysis, information on the choice of priors and Markov chain Monte Carlo settings
☒	☐	For hierarchical and complex designs, identification of the appropriate level for tests and full reporting of outcomes
☐	☒	Estimates of effect sizes (e.g. Cohen's d, Pearson's r), indicating how they were calculated

Our web collection on statistics for biologists contains articles on many of the points above.

Software and code

Policy information about availability of computer code

Data collection	software used was an in house pipeline. we used RDP classifier 2.12 with SILVA 16S rRNA database (version 128) for taxonomic calling.
Data analysis	data were analyzed in R version 3.6. The following packages were used for analysis: VEGAN (for alpha-diversity calculation and beta diversity), RandomForerst and MR.

For manuscripts utilizing custom algorithms or software that are central to the research but not yet described in published literature, software must be made available to editors and reviewers. We strongly encourage code deposition in a community repository (e.g. GitHub). See the Nature Research guidelines for submitting code & software for further information.

Data

Policy information about availability of data

All manuscripts must include a data availability statement. This statement should provide the following information, where applicable:

- Accession codes, unique identifiers, or web links for publicly available datasets
- A list of figures that have associated raw data
- A description of any restrictions on data availability

The source data for for table 1 and 2 and supplementary tables are provided with the paper. the data supporting the findings of this study are not allowed to be published in an open or closed data repositories due to the General Data Protection Regulation (GDPR). However, the data can be made available upon reasonable request. For request regarding the Rotterdam Study microbiome dataset please contact Frank van Rooij (f.vanrooij@erasmusmc.nl).

Field-specific reporting

Please select the one below that is the best fit for your research. If you are not sure, read the appropriate sections before making your selection.

[x] Life sciences [ ] Behavioural & social sciences [ ] Ecological, evolutionary & environmental sciences

For a reference copy of the document with all sections, see nature.com/documents/nr-reporting-summary-flat.pdf

Life sciences study design

All studies must disclose on these points even when the disclosure is negative.

Sample size	No sample size was calculated. Sample size was selected based on availability of the data, i.e., number of participants in the Rotterdam Study (RS-III-2) of whom microbiome and depression scores were taken.
Data exclusions	participants who used antidepressants were excluded. participants were excluded whom samples were more than 3 days on travel to reach the Erasmus MC. also samples with less than 10,000 reads were also excluded.
Replication	the associations were replicated in an independent cohort (n=1,539)
Randomization	not relevant: we have an independent cohort for replication.
Blinding	there was no group allocation in our study. individuals who have done the sample collection and sample analysis did not perform the statistical analysis.

Reporting for specific materials, systems and methods

We require information from authors about some types of materials, experimental systems and methods used in many studies. Here, indicate whether each material, system or method listed is relevant to your study. If you are not sure if a list item applies to your research, read the appropriate section before selecting a response.

Materials & experimental systems

n/a	Involved in the study	
[x]	[ ]	Antibodies
[x]	[ ]	Eukaryotic cell lines
[x]	[ ]	Palaeontology and archaeology
[x]	[ ]	Animals and other organisms
[ ]	[x]	Human research participants
[ ]	[ ]	Clinical data
[x]	[ ]	Dual use research of concern

Methods

n/a	Involved in the study	
[x]	[ ]	ChIP-seq
[x]	[ ]	Flow cytometry
[x]	[ ]	MRI-based neuroimaging

Human research participants

Policy information about studies involving human research participants

Population characteristics	Rotterdam Study (RS). cohort III. visit 2 microbiome set (n=1,054; 590 females and 464 males). cohort charactristics=average (standard deviation): age=56 (5.9), BMI=27 (4.4), alcohol(glass/day)=1.3(2.6), depression score=4.7 (6.2), smoking (current, ever, never)=137,533,384.
Recruitment	RS-III: recruitment of individuals aged > 45. living tin the Ommord district, and not already included in RS-I and RS-II.
Ethics oversight	Medical Ethical Committee of Erasmus MC (University Medical Center Rotterdam, The Netherlands, MEC02.1015)

Note that full information on the approval of the study protocol must also be provided in the manuscript.

Clinical data

Policy information about clinical studies

All manuscripts should comply with the ICMJEguidelines for publication of clinical research and a completedCONSORT checklist must be included with all submissions.

Clinical trial registration	*Provide the trial registration number from ClinicalTrials.gov or an equivalent agency.*
Study protocol	*Note where the full trial protocol can be accessed OR if not available, explain why.*

Data collection	*Describe the settings and locales of data collection, noting the time periods of recruitment and data collection.*
Outcomes	*Describe how you pre-defined primary and secondary outcome measures and how you assessed these measures.*
